# Karyotype analysis of seven species of the tribe Lophiohylini (Hylinae, Hylidae, Anura), with conventional and molecular cytogenetic techniques

**DOI:** 10.3897/CompCytogen.v6i4.3945

**Published:** 2012-12-03

**Authors:** Simone Lilian Gruber, Célio Fernando Baptista Haddad, Sanae Kasahara

**Affiliations:** 1 UNESP, Universidade Estadual Paulista, Instituto de Biociências, Departamento de Biologia, Av. 24A, 1515, 13506-900, Rio Claro, SP, Brasil; 2 UNESP, Universidade Estadual Paulista, Instituto de Biociências, Departamento de Zoologia, Av. 24A, 1515, 13506-900, Rio Claro, SP, Brasil

**Keywords:** Amphibian cytogenetics, Ag-NOR, C-banding, rDNA probe, telomeric probe, fluorochrome staining

## Abstract

Few species of the tribe Lophiohylini have been karyotyped so far, and earlier analyses were performed mainly with standard staining. Based on the analysis of seven species with use of routine banding and molecular cytogenetic techniques, the karyotypes were compared and the cytogenetic data were evaluated in the light of the current phylogenies. A karyotype with 2n = 24 and NOR in the chromosome 10 detected by Ag-impregnation and FISH with an rDNA probe was shared by *Aparasphenodon bokermanni* Miranda-Ribeiro, 1920, *Itapotihyla langsdorffii* (Duméril and Bibron, 1841), *Trachycephalus* sp., *Trachycephalus mesophaeus* (Hensel, 1867), and *Trachycephalus typhonius* (Linnaeus, 1758). *Phyllodytes edelmoi* Peixoto, Caramaschi et Freire, 2003 and *Phyllodytes luteolus* (Wied-Neuwied, 1824) had reduced the diploid number from 2n = 24 to 2n = 22 with one of the small-sized pairs clearly missing, and NOR in the large chromosome 2, but the karyotypes were distinct regarding the morphology of chromosome pairs 4 and 6. Based on the cytogenetic and phylogenetic data, it was presumed that the chromosome evolution occurred from an ancestral type with 2n = 24, in which a small chromosome had been translocated to one or more unidentified chromosomes. Whichever hypothesis is more probable, other rearrangements should have occurred later, to explain the karyotype differences between the two species of *Phyllodytes* Wagler, 1830. The majority of the species presented a small amount of centromeric C-banded heterochromatin and these regions were GC-rich. The FISH technique using a telomeric probe identified the chromosome ends and possibly (TTAGGG)_n_-like sequences in the repetitive DNA out of the telomeres in *Itapotihyla langsdorffii* and *Phyllodytes edelmoi*. The data herein obtained represent an important contribution for characterizing the karyotype variability within the tribe Lophiohylini scarcely analysed so far.

## Introduction

The hylids of the subfamily Hylinae Rafinesque, 1815 are grouped into four large tribes: Cophomantini, Dendropsophini, Hylini, and Lophiohylini (Faivovich et al. 2005, Wiens et al. 2010). In the tribe Lophiohylini 11 genera are assigned and the majority of them included the known casque-headed frogs which are distributed throughout Central and South America. According to Faivovich et al. (2005), despite the phylogenetic review based mainly on molecular gene sequencing, few morphological synapomorphies support the current taxonomy of the tribe Lophiohylini and many unresolved questions still remain. Recently, the separate genus *Phytotriades* Jowers, Downieb et Cohen, 2009 was erected for the species *Phyllodytes auratus* (Boulenger, 1917) based on analysis of mitochondrial rDNA sequences.

About 70 species are recognised in the tribe Lophiohylini (Frost 2011), but only a dozen of them from seven genera have been karyotyped (Catroli and Kasahara 2009). Earlier analyses, performed exclusively with standard staining, were conducted during the 1960s and 1970s in the species *Aparasphenodon brunoi* Miranda-Ribeiro, 1920, *Itapotihyla langsdorffii* (Duméril & Bibron, 1841), *Osteopilus septentrionalis* (Duméril & Bibron, 1841), *Trachycephalus mesophaeus* (Hensel, 1867), and *Trachycephalus typhonius* (Linnaeus, 1758), all of them with 2n = 24, and *Osteopilus brunneus* Trueb and Tyler, 1974 with 2n = 34 (Duellman and Cole 1965, Rabello 1970, Bogart and Bogart 1971, Foresti 1972, Bogart 1973, Cole 1974). Subsequently studies were carried out with use of banding and FISH techniques on some of these species (*Aparasphenodon brunoi*, *Itapotihyla langsdorffii*, *Osteopilus septentrionalis*, and *Osteopilus brunneus*) and also in *Argenteohyla siemersi* (Mertens, 1937), *Corythomantis greeningi* Boulenger, 1896, *Osteocephalus taurinus* Steindachner, 1862, *Osteopilus dominicensis* (Tschudi, 1838), and *Osteopilus marianae* (Dunn, 1926), all of them with 2n = 24, and in *Osteopilus wilderi* (Dunn, 1925) with 2n = 28 (Schmid 1978, 1980, Anderson 1996, Morand and Hernando 1996, Kasahara et al. 2003, Nunes and Fagundes 2008). The species of the Lophiohylini genera *Nyctimantis* Boulenger, 1882, *Tepuihyla* Ayarzagüena, Señaris and Gorzula, 1993, *Phyllodytes* Wagler, 1830, and *Phytotriades* Jowers, Downieb et Cohen, 2008 have never been karyotyped.

The present paper deals with the chromosome analysis of *Aparasphenodon bokermanni* Pombal, 1993, *Itapotihyla langsdorffii*, *Phyllodytes edelmoi* Peixoto, Caramaschi & Freire, 2003, *Phyllodytes luteolus* (Wied-Neuwied, 1824), *Trachycephalus mesophaeus*, *Trachycephalus typhonius*, and *Trachycephalus* sp. (probably an undescribed species) with use of routine and molecular cytogenetic techniques. The aim was to analyze species never karyotyped before and to improve the cytogenetic data from some other species, in order to better characterizing the karyotype variability within the tribe Lophiohylini and to carry out a more comprehensive comparative analysis in the light of the current phylogeny.

## Material and methods

Cytogenetic analyses were performed with specimens of *Aparasphenodon* Miranda-Ribeiro, 1920, *Itapotihyla* Faivovich, Haddad, Garcia, Frost, Campbell, et Wheeler, 2005, *Phyllodytes*, and *Trachycephalus* Tschudi, 1838 (Table 1) collected in the Brazilian states of Alagoas (AL), Bahia (BA), Espírito Santo (ES), Mato Grosso (MS), and São Paulo (SP). The voucher specimens were deposited in the amphibian collection Célio Fernando Baptista Haddad (CFBH), housed in the Departamento de Zoologia, UNESP, Rio Claro, SP, Brazil.

**Table 1.** Species, number of individuals, sex, voucher numbers, and collection locations in Brazil.

**species**	**number**	**sex**	**voucher numbers CFBH**	**collection locations**
*Aparasphenodon bokermanni*	1	male	22575	Cananéia, SP (25°01'19"S, 47°55'41"W)
*Itapotihyla langsdorffii*	2	males	22369, 22370	Ilhéus, BA (14°47'29"S, 39°02'41"W)
	1	female	30973	Rio Claro, SP (22°25'20"S, 47°34'23"W)
*Phyllodytes edelmoi*	2	females	22583, 22584	Maceió, AL (09°40'06"S, 35°43'59"W)
	1	male	22585	Maceió, AL (09°40'06"S, 35°43'59"W)
*Phyllodytes luteolus*	2	males	22462, 22463	Guaraparí, ES (20°39'01"S, 40°29'10"W)
*Trachycephalus* sp.	1	male	20664	Paranaíta, MT (09°40'56"S, 56°28'50"W)
*Trachycephalus mesophaeus*	3	males	22366, 22367, 22368	Ilhéus, BA (14°47'29"S, 39°02'41"W)
	2	females	22371, 22372	Ilhéus, BA (14°47'29"S, 39°02'41"W)
	1	juvenile	22484	Ubatuba, SP (23°26'19"S, 45°05'25"W)
	1	male	24222	Biritiba Mirim, SP (23°34'17"S, 46°02'15"W)
*Trachycephalus typhonius*	1	female	22365	Porto Primavera, MS (22°26'01"S, 52°58'11"W)
	1	male	10033	Rio Claro, SP (22°25'20"S, 47°34'23"W)

CFBH - Célio Fernando Baptista Haddad Collection, UNESP, Rio Claro, SP, Brazil.

Direct cytological suspensions of bone marrow, liver, and testes were prepared according to the procedures described in Baldissera et al. (1993), and from the intestinal epithelium according to the method of Schmid (1978). The slides were subjected to standard Giemsa staining and to the techniques of Ag-NOR (Howell and Black 1980), C-banding (Sumner 1972), and double staining with the fluorochromes AT-specific DAPI and GC-specific CMA_3_ (Christian et al. 1998). Fluorescent in situ hybridisation (FISH) (Pinkel et al. 1986) was carried out using the ribosomal probe HM123 (Meunier-Rotival et al. 1979) and a telomeric probe (TTAGGG)_n_ according to the DAKO kit instructions (Denmark). The Ag-NOR technique was frequently performed using the same slide after Giemsa staining or FISH technique with the HM123 probe. In both cases, the slides were washed with xylol to remove the immersion oil and then submitted to the technique for obtaining Ag-NOR as usual but decreasing the time of incubation in all steps of the procedure. Chromosomal images were captured with an Olympus digital camera D71 with use of the DP Controller program. The bi-armed chromosomes were classified as metacentric, submetacentric or subtelocentric according to the nomenclature proposed by Green and Sessions (1991, 2007).

## Results

**Figure 1.**
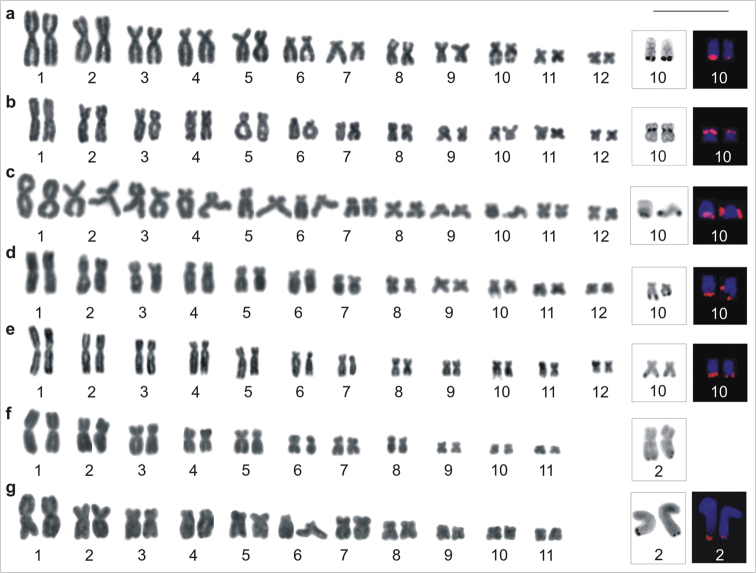
Giemsa-stained karyotypes. **a** *Aparasphenodon bokermanni*, male, 2n = 24 **b** *Itapotihyla langsdorffii*, male, 2n = 24 **c** *Trachycephalus* sp., male, 2n = 24 **d** *Trachycephalus mesophaeus*, male, 2n = 24 **e** *Trachycephalus typhonius*, male, 2n = 24; f. *Phyllodytes edelmoi*, male, 2n = 22 **g** *Phyllodytes luteolus*, male, 2n = 22. The insets show the chromosome pairs with Ag-NOR and FISH using the rDNA probe. Bar = 10 mm.

Specimens of *Aparasphenodon bokermanni*, *Itapotihyla langsdorffii*, *Trachycephalus* sp., *Trachycephalus mesophaeus*, and *Trachycephalus typhonius* had a diploid number of 2n = 24 (Fig. 1a–e) and a fundamental number FN = 48 and *Phyllodytes edelmoi* and *Phyllodytes luteolus* had 2n = 22, FN = 44 (Fig. 1f–g). The Table 2 presents the relative length (RL), centromeric index (CI), and the centromeric position (CP) with morphologic classification of the chromosomes of the seven species.

**Table 2.** Relative length (RL), centromeric index (CI), and nomenclature for centromeric position (CP) on mitotic chromosomes according to Green and Sessions (1991, 2007).

**Species**		**Chromosome number**											
		**1**	**2**	**3**	**4**	**5**	**6**	**7**	**8**	**9**	**10**	**11**	**12**
*Aparasphenodon bokermanni*	RL	15.57	12.93	10.65	9.63	9.48	7.48	5.68	6.78	6.27	6.5	4.12	3.83
	CI	0.479	0.459	0.396	0.263	0.344	0.286	0.321	0.464	0.487	0.284	0.420	0.465
	CP	**m**	**m**	**m**	**sm**	**sm**	**sm**	**sm**	**m**	**m**	**sm**	**m**	**m**
*Itapotihyla langsdorffii*	RL	15.06	13.52	11.50	10.41	9.82	7.68	6.59	6.35	5.17	5.00	5.02	3.90
	CI	0.460	0.421	0.355	0.241	0.361	0.225	0.391	0.483	0.472	0.472	0.460	0.467
	CP	**m**	**m**	**sm**	**st**	**sm**	**st**	**m**	**m**	**m**	**m**	**m**	**m**
*Trachycephalus* sp.	RL	14.57	11.79	11.57	9.95	9.18	7.81	6.75	6.06	4.43	5.15	4.65	4.30
	CI	0.430	0.429	0.383	0.257	0.319	0.261	0.344	0.453	0.456	0.301	0.443	0.461
	CP	**m**	**m**	**m**	**sm**	**sm**	**sm**	**sm**	**m**	**m**	**sm**	**m**	**m**
*Trachycephalus mesophaeus*	RL	14.33	13.57	10.66	10.47	9.04	7.98	6.76	6.35	5.94	6.97	4.72	3.83
	CI	0.457	0.435	0.366	0.268	0.370	0.224	0.338	0.481	0.424	0.351	0.353	0.414
	CP	**m**	**m**	**sm**	**sm**	**sm**	**st**	**sm**	**m**	**m**	**sm**	**sm**	**m**
*Trachycephalus typhonius*	RL	15.63	12.80	11.05	10.51	10.06	8.16	7.07	6.02	5.02	5.23	4.59	4.10
	CI	0.462	0.397	0.364	0.236	0314	0.200	0.317	0.424	0.444	0.304	0.461	0.485
	CP	**m**	**m**	**sm**	**st**	**sm**	**st**	**sm**	**m**	**m**	**sm**	**m**	**m**
*Phyllodytes edelmoi*	RL	18.38	13.74	12.88	9.90	9.74	7.73	6.86	6.77	4.88	4.06	3.74	--
	CI	0.453	0.403	0.335	0.430	0.341	0.414	0.367	0.404	0.440	0.444	0.472	--
	CP	**m**	**m**	**sm**	**m**	**sm**	**m**	**sm**	**m**	**m**	**m**	**m**	**--**
*Phyllodytes luteolus*	RL	16.62	12.56	11.11	10.65	9.57	8.72	8.38	7.05	5.38	4.80	4.56	--
	CI	0.450	0.422	0.370	0.249	0.352	0.237	0.336	0.354	0.472	0.430	0.443	--
	CP	**m**	**m**	**sm**	**st**	**sm**	**st**	**sm**	**sm**	**m**	**m**	**m**	**--**

m = metacentric; sm = submetacentric; st = subtelocentric.

The technique of Ag-NOR was carried out in all species. In the 2n = 24 karyotypes the Ag-NORs were located on chromosome 10, at the terminal long arm in the case of *Aparasphenodon bokermanni*, *Trachycephalus* sp., *Trachycephalus mesophaeus*, and *Trachycephalus typhonius* (Fig. 1a, c–e), or at the interstitial short arm in *Itapotihyla langsdorffii* (Fig. 1b). In *Phyllodytes edelmoi* and *Phyllodytes luteolus* Ag-NOR was located at the terminal long arm of chromosome 2 (Fig. 1f–g). The Ag-impregnation occurred in the sites of the secondary constriction, although this marker was not always visualised in the standard stained chromosomes. In *Aparasphenodon bokermanni* and *Trachycephalus* sp. and in some individuals of the remaining species, there was variation in the pattern of Ag-NOR labelling. Within the same individual, metaphases exhibited Ag-NORs with conspicuous or slight difference in size or carried two Ag-NORs with equivalent sizes; occasionally a single Ag-NOR per metaphase was also observed in the same cytological preparation. FISH with an rDNA probe was performed in six species, with exception of *Phyllodytes edelmoi*. Two fluorescent signals were observed in all analysed metaphases (Fig. 1a–e, g). In the species *Trachycephalus* sp. and *Trachycephalus mesophaeus* the hybridisation signals always presented the same size and in *Aparasphenodon bokermanni*, *Itapotihyla langsdorffii*, *Trachycephalus typhonius*, and *Phyllodytes luteolus* the labelling was heteromorphic in all metaphases.

**Figure 2.**
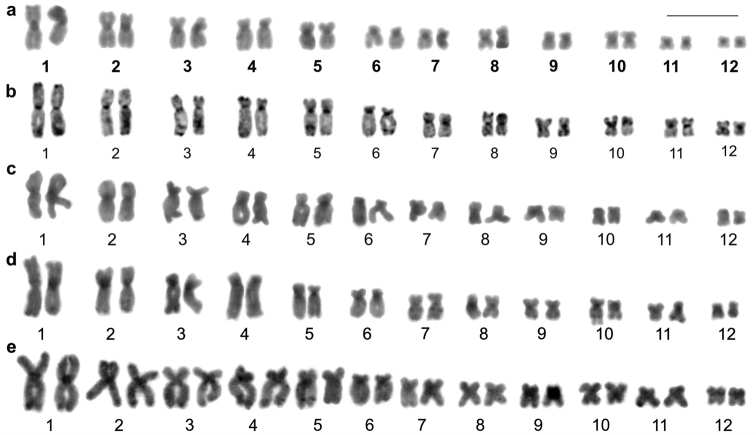
C-banded karyotypes. **a** *Aparasphenodon bokermanni* **b** *Itapotihyla langsdorffii* **c** *Trachycephalus* sp. **d** *Trachycephalus mesophaeus* **e** *Trachycephalus typhonius*. Bar = 10 mm.

**Figure 3.**
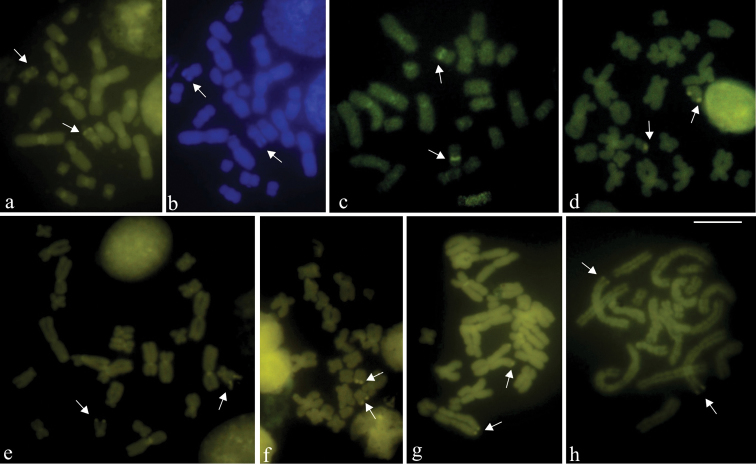
Fluorochrome-stained metaphases. **a, c-h** CMA_3_ **b** DAPI **a–b** *Aparasphenodon bokermanni* **c** *Itapotihyla langsdorffii* **d** *Trachycephalus* sp. **e** *Trachycephalus mesophaeus* **f** *Trachycephalus typhonius* **g** *Phyllodytes edelmoi* **h** *Phyllodytes luteolus*. Bright DAPI fluorescence at the terminal short arms of chromosomes 10 (arrows) and the negative centromeric region are shown in **a**. CMA_3_ fluorescent labelling of the NOR site (arrows) and in the centromeric region of chromosomes in **a**, **c–h**. Bar = 10 mm.

The C-banding in *Aparasphenodon bokermanni*, *Itapotihyla langsdorffii*, *Trachycephalus* sp., *Trachycephalus mesophaeus*, and *Trachycephalus typhonius* showed heterochromatin distribution in the pericentromeric regions of all chromosomes (Fig. 2). In *Itapotihyla langsdorffii* additional C-bands were noticed at terminal (chromosomes 1 and 4) and interstitial (chromosome 5) regions. This technique was carried out in mitotic and meiotic cytological preparations of *Phyllodytes edelmoi* and *Phyllodytes luteolus*, but no C-banded region was demonstrated in the chromosomes of these species. The NOR site in all species was brilliant with CMA_3_, as well as the chromosome pericentromeric region (Fig. 3a, c–h). The pericentromeric fluorescence was in general faint and not visualised in all chromosomes. In *Aparasphenodon bokermanni* the centromeric signals were particularly prominent in size and brightness (Fig. 3a). No brilliant labelling was observed after DAPI staining in any species, except in *Aparasphenodon bokermanni* which showed slight fluorescence at the terminal short arm of chromosome 10 (Fig. 3b). The chromosome pericentromeric region of this species was DAPI-negative.

**Figure 4.**
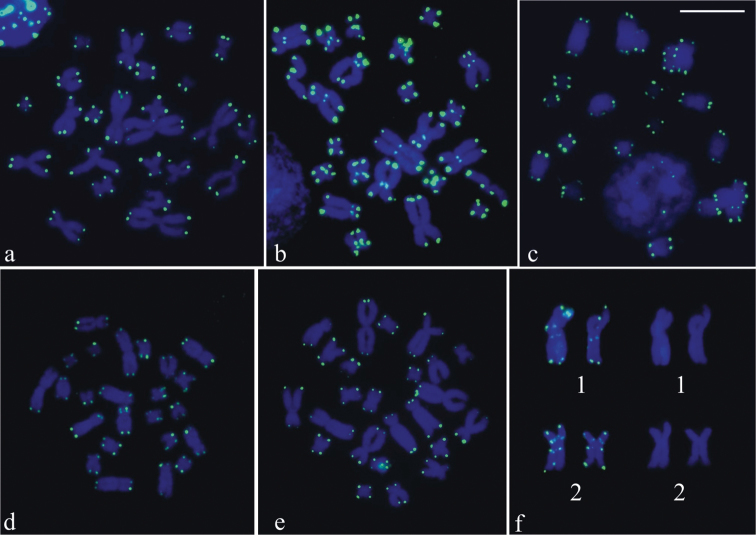
FISH using a telomeric probe. **a** *Aparasphenodon bokermanni* **b** *Itapotihyla langsdorffii* **c** *Trachycephalus* sp. **d,** *Trachycephalus mesophaeus* **e** *Trachycephalus typhonius* **f** *Phyllodytes edelmoi*. In **b** hybridisation labelling is visible in the centromeric region of the chromosomes and in **f,** at the proximal short and long arms of chromosomes 1 and 2 observed with telomeric probe hybridisation (left) and with DAPI staining (right). Bar = 10 mm.

The telomeric probe hybridized on the chromosome ends in six of the species, excepting in *Phyllodytes luteolus* without cytological material available for the FISH technique. Figure 4a–e showed metaphases of *Aparasphenodon bokermanni*, *Itapotihyla langsdorffii*, *Trachycephalus* sp., *Trachycephalus mesophaeus*, and *Trachycephalus typhonius* with probe labelling at the chromosome ends and, in the case of *Itapotihyla langsdorffii* (Fig. 4b), also in the pericentromeric region. In *Phyllodytes edelmoi* no good metaphases were obtained, but the chromosomes showed telomeric labelling. In one metaphase of this species, however, the large-sized chromosome pair 1 and 2 had probe hybridization at the proximal short and long arms (Fig. 4f).

**Figure 5.**
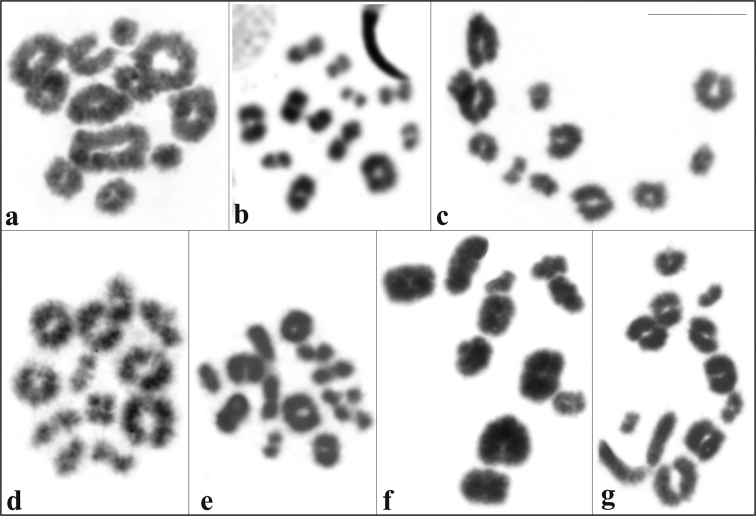
Giemsa-stained diakinesis and metaphases I cells. **a** *Aparasphenodon bokermanni*, 2n = 24 **b** *Itapotihyla langsdorffii*, 2n = 24 **c** *Trachycephalus* sp., 2n = 24 **d** *Trachycephalus mesophaeus*, 2n = 24 **e** *Trachycephalus typhonius*, 2n = 24 **f** *Phyllodytes edelmoi*, 2n = 22 **g** *Phyllodytes luteolus*, 2n = 22. Bar = 10 mm.

No sex-chromosome pairs were detected in male or female specimens of *Itapotihyla langsdorffii*, *Trachycephalus mesophaeus*, *Trachycephalus typhonius*, and *Phyllodytes edelmoi*. In the remaining three species only males were karyotyped with no evidence of sex related heteromorphism. Meiotic analysis confirmed the diploid number in all species (Fig. 5a–g). *Aparasphenodon bokermanni*, *Itapotihyla langsdorffii*, *Trachycephalus* sp., *Trachycephalus mesophaeus*, and *Trachycephalus typhonius* showed 12 bivalents. *Phyllodytes edelmoi* and *Phyllodytes luteolus* showed 11 bivalents.

The main cytogenetic data obtained in the present study are summarized in the Table 3.

**Table 3.** Data on chromosome number, chromosome formula, NOR and telomeric sequence localization, C-band distribution and molecular content of repetitive DNA sequences of studied species.

**species**	**2n**	**fomula**	NOR	**Tel**	**C bands**	**DAPI**	**CM_A_3**
*Aparasphenodon bokermanni*	24	7m+5sm	11qt	T	C+NOR	10pt	C*+NOR
*Itapotihyla langsdorffii*	24	8m+2sm+2st	11pi	T+C	C+NOR	--	C+NOR
*Trachycephalus* sp.	24	7m+5sm	11qt	T	C+NOR	--	C+NOR
*Trachycephalus mesophaeus*	24	5m+6sm+1st	11qt	T	C+NOR	--	C+NOR
*Trachycephalus typhonius*	24	6m+4sm+2st	11qt	T	C+NOR	--	C+NOR
*Phyllodytes edelmoi*	22	8m+3sm	2qt	T+C	C+NOR	--	C+NOR
*Phyllodytes luteolus*	22	5m+4sm+2st	2qt	---	C+NOR	--	C+NOR

m = metacentric; sm = submetacentric; st = subtelocentric; p = short chromosome arm; q = long chromosome arm; i = interstitial region; t = terminal region; T = telomere; C = centromeric/ pericentromeric region; * intense mark.

## Discussion

The species of the tribe Lophiohylini *Aparasphenodon bokermanni*, *Itapotihyla langsdorffii*, *Trachycephalus* sp., *Trachycephalus mesophaeus*, and *Trachycephalus typhonius* with 2n = 24 shared indistinguishable karyotypes even though there was discrepancy in morphological classification shown in Table 2 for some chromosomes, as the chromosome 3 of the species, due to slight differences in the chromosome arm proportion. No evidence of population karyotype difference was observed for *Itapotihyla langsdorffii*, *Trachycephalus mesophaeus*, and *Trachycephalus typhonius* sampled in distinct locations. Considering previous data for these three species (Rabello 1970, Foresti 1972, Bogart 1973, Kasahara et al. 2003, Nunes and Fagundes 2008), no difference was noticeable in the karyotypes, although the morphological classification of chromosomes and the ordering of the pairs in the distinct karyograms were not the same.

The chromosome constitution with 2n = 24 herein described is the same as found for the remaining eight species of Lophiohylini analysed so far, corresponding to *Aparasphenodon brunoi*, *Argenteohyla siemersi*, *Corythomantis greeningi*, *Osteocephalus taurinus*, *Osteopilus dominicensis*, *Osteopilus marianae*, *Osteopilus septentrionalis*, and an unidentified species of *Trachycephalus* (see Catroli and Kasahara 2009 for references). This finding suggests a high degree of karyotype conservation within the tribe. Actually, a detailed comparative analysis of the replication banding obtained by BrdU incorporation had shown unequivocal homeology at least among the chromosomes of *Aparasphenodon brunoi*, *Corythomantis greeningi*, and *Itapotihyla langsdorffii* (Kasahara et al. 2003). It is important to emphasise that this conservative pattern of chromosome constitution has been observed in representatives of Hylinae and, according to the molecular phylogeny of Faivovich et al. (2005), a karyotype with 2n = 24 could be a synapomorphic condition within this subfamily. Another karyotype characteristic shared by the majority of the Lophiohylini species with 2n = 24 is the NOR site in a small-sized chromosome (Schmid 1978, 1980, Anderson 1996, Kasahara et al. 2003, Nunes and Fagundes 2008), with the exception of *Argenteohyla siemersi* (Morand and Hernando 1996) with NOR in the chromosome pair 4.

*Phyllodytes edelmoi* and *Phyllodytes luteolus*, the first two species of the genus that were analysed so far, had reduced the diploid numbers from 2n = 24 to 2n = 22 and the NOR site was in the large-sized chromosome 2. Nevertheless, the karyotypes of these two species were distinct regarding the morphology of pairs 4 and 6, that is, in *Phyllodytes edelmoi* these pairs were metacentric and in *Phyllodytes luteolus* they were subtelocentric, as it has been usually observed in Hylinae species with 2n = 24. The discrepancy in the morphology of the chromosome pairs 4 and 6 was supported by the chromosome measurements and the mechanism responsible for these differences might be, for example, a pericentric inversion or another type of chromosome rearrangement, but this could not be determined at least with the cytogenetic techniques used here.

Within the sub-family Hylinae, variation as resulted of fusion events from an ancestral karyotype with 24 chromosomes was described for *Hypsiboas albopunctatus* (Spix, 1824) (2n = 22) and for species of the genus *Aplastodiscus* (2n = 18, 20, 22) (Gruber et al. 2007, Gruber et al. 2012). Although the chromosomes involved in the rearrangements could not be recognized with certainty in neither case, the derived chromosomes in *Hypsiboas albopunctatus* and in *Aplastodiscus* species were tentatively identified by their altered morphology regarding the presumed ancestral. The reduction in the diploid number to 2n = 22 in *Phyllodytes* might also be due to fusion rearrangement of end-to-end or centric type from the ancestral 2n = 24 karyotype. Taking into account that the two analysed species presented four small pairs instead of five and the NOR was on large-sized pair, the fusion, at first sight, occurred between a small NOR-bearing chromosome and chromosome 2. Nevertheless, the NOR-bearing chromosome 2 of *Phyllodytes* had no noticeable relative size differences regarding the chromosome 2, not carrying NOR, of the 2n = 24 species. Another possibility is the translocation of one of the smallest chromosomes to chromosome 1, since this element in the *Phyllodytes* species has a larger relative length when compared to the chromosome 1 of 2n = 24 karyotypes. The translocation of a small pair to more than one unidentified chromosomes, leading to the reduction in the diploid number to 2n = 22 could not be discarded. Whichever of the hypotheses is more probable, other rearrangements should have occurred later, to explain the differences observed between the karyotypes of the two species of *Phyllodytes*. Certainly, additional cytogenetic analyses within the genus are necessary to outline the events occurred during the chromosome evolution.

In males and females of *Itapotihyla langsdorffii*, *Phyllodytes edelmoi*, *T mesophaeus*, and *Trachycephalus typhonius* and in males of *Aparasphenodon bokermanni*, *Trachycephalus* sp., and *Phyllodytes luteolus* heteromorphic sex chromosomes were not observed. Nevertheless in females of these three latter species sex chromosomes could not be discarded. Anurans, in general, do not present cytological sex chromosome differentiation and both male or female heterogamety has been described in some species (Schmid et al. 2010).

A single NOR pair located in a small-sized chromosome (Schmid 1978, Green and Sessions 2007, Schmid et al. 2010) is a shared characteristic for the majority of the Lophiohylini species and this condition has also been frequently observed in other Hylinae of the genera *Bokermannohyla* Faivovich, Haddad, Garcia, Frost, Campbell & Wheeler, 2005, *Hyla* Laurenti, 1768, *Hypsiboas* Wagler, 1830, and *Scinax* Wagler, 1830 (clade *Scinax ruber*) (Catroli et al. 2011, Cardozo et al. 2011). Although the NOR-bearing pair has been referred in the literature to as chromosome pairs 10, 11, or 12, most probably we are dealing with the same pair. In fact, Kasahara et al. (2003) demonstrated close correspondence in the replication banding patterns between the NOR-bearing chromosomes 10 of the Lophiohylini *Aparasphenodon brunoi*, *Corythomantis greeningi*, and *Itapotihyla langsdorffii* and the NOR-bearing chromosomes 12 of the Dendropsophini *Scinax fuscovarius* (Lutz, 1925). As stressed by Cardozo et al. (2011), NOR in a small-sized chromosome is considered a plesiomorphy within the subfamily, wherefore NOR location out of small element, as observed in *Argenteohyla siemersi* and in *Phyllodytes*, is a derived condition.

The NOR marker chromosome in our species of Lophiohylini with 2n = 24 was considered as the 10 and the rDNA sequences were at the interstitial short arm or at the terminal long arm, but no major differences were observed in the morphology of the chromosomes 10 among distinct species. Therefore, the mechanism that changed the NOR sites apparently was not a gross rearrangement, but minute structural rearrangements or transposition by means of mobile elements could not be discarded. If the movement of the NOR from chromosome 10 to chromosome 2 in *Phyllodytes* species was not a direct consequence of the rearrangement which reduced the diploid number in the genus, one of the two mentioned mechanisms would also be a reasonable explanation for the discrepant NOR site, in *Phyllodytes edelmoi* and in *Phyllodytes luteolus*.

The technique of Ag-impregnation showed large variation in the Ag-NOR pattern within the same individual. Nevertheless, the FISH with an rDNA probe revealed that the NOR labelling in each individual had either equivalent or distinct size in all the analysed cells. Such data allowed us to conclude that most probably the Ag-NOR variation was a result of differential activity of ribosomal gene in *Trachycephalus* sp. and *Trachycephalus mesophaeus* because the hybridization labelling had the same size in both homologues; on the other hand, different amounts of repetitive rDNA units would be responsible for the observed Ag-NOR variation in *Aparasphenodon bokermanni*, *Itapotihyla langsdorffii*, and *Trachycephalus typhonius* because hybridization labelling had distinct sizes in both homologues. The single Ag-NOR seen occasionally in some metaphases could be attributed to the lacking or insufficient amount of the non-histone proteins available for the Ag-impregnation.

The chromosomes of the species herein analysed produced C-banding results only after over treatment of the distinct steps of the technique. However, it was undoubtedly demonstrated that heterochromatin was distributed mainly in the centromeric regions. A similar centromeric C-banding pattern had been described in *Aparasphenodon brunoi*, *Corythomantis greeningi*, and *Itapotihyla langsdorffii* (Kasahara et al. 2003) besides some interstitial and terminal additional C-bands in the latter species. The lack of C-bands in the chromosomes of *Phyllodytes edelmoi* and *Phyllodytes luteolus* might be due to the absence of repetitive DNA identifiable by means of C-banding technique. Nevertheless, it will be important to confirm such possibility or if we are dealing with some technical difficulty, since CMA_3_ staining at the centromeres in both species, albeit with faint fluorescence, confirmed the presence of repetitive sequences in these regions.

Surprisingly, in spite of the low amount of C-banded heterochromatin, *Aparasphenodon bokermanni* showed conspicuous bright fluorescence at the centromeres, similar to that observed in *Aparasphenodon brunoi* (Kasahara et al. 2003). This result and the corresponding DAPI-negative fluorescence in both species indicated presence of a particular repetitive DNA characteristic of the genus *Aparasphenodon* with an exceptional GC-content. Besides the centromere, each of these two species had own fluorescent markers in other chromosome regions: *Aparasphenodon brunoi* exhibited a bright CMA_3_ site in the long arm of chromosome 5 (Kasahara et al. 2003), whereas *Aparasphenodon bokermanni* had bright CMA_3_ site in the long arm of chromosome 10 and bright DAPI site in the short arm of the same chromosome 10. *Itapotihyla langsdorffii* and the species of *Trachycephalus* and *Phyllodytes* showedfaint centromeric fluorescence with CMA_3_ indicating that the GC-content was not high.

Although the FISH with the telomeric probe is primarily designed for identification of chromosome-ends, this procedure may provide information about the molecular nature of some repetitive sequences. As far as it has been shown, distinct organisms, including frogs (Meyne et al. 1990, Wiley et al. 1992, Nanda et al. 2008, Gruber et al. 2012), disclosed hybridization out of the telomeres, even in the cases without evidence of chromosome rearrangements. This would mean presence of telomere-like sequences (TTAGGG)_n_ in sites of repetitive DNA and it seems to explain the labelling out the telomere sites in *Itapotihyla langsdorffii* and *Phyllodytes edelmoi*. These data reinforce the importance of the FISH with the telomeric probe used in combination with base-specific fluorochrome staining and C-banding for obtaining information on the content of distinct repetitive regions.

The interstitial hybridization signals of telomeric probe could correspond to vestiges of true telomeres, as reported in rodents (Fagundes et al. 1997, Ventura et al. 2006), but in our sampled species, there was no evidence of telomere remnants resulted probably from chromosome rearrangements. Despite presumed fission and fusion during the chromosome evolution, Anderson (1996) noticed no hybridisation interstitial labelling in the Lophiohylini *Osteopilus septentrionalis* (2n=24) and *Osteopilus brunneus* (2n=34).

Based on the data of 22 species, a phylogenetic tree of the Lophiohylini was provided by Faivovich et al. (2005). Later, Jowers et al. (2008) added the molecular information of *Phytotriades auratus* and, more recently, the phylogeny of Lophiohylini was expanded by Wiens et al. (2010) for a total of 35 representatives. All these trees support the monophyly of the tribe, although the relationships of the distinct genera remain unclear. In the phylogeny of Faivovich et al. (2005) *Phyllodytes* appears in an isolated clade at a basal position. In the phylogeny of Wiens et al. (2010) the representatives of Lophiohylini are grouped into two major sister-clades and the species of *Phyllodytes* and *Osteopilus* are included in one of these clades, along with the species with 2n =24. Regardless of which of the two phylogenetic hypotheses is most accurate, it is clear that 2n = 22 exhibited by the species of *Phyllodytes* is a derived condition.

The present study showed that in spite of the high similarity of the chromosome constitution and of the NOR pattern among the species of Lophiohylini with 2n = 24, the karyotypes could be recognized by the nature of the repetitive sequences, as differentiated through C-banding, base-specific fluorochrome staining, and, in a certain extension, by FISH with telomeric probe. Cytogenetic information on the tribe is still minimal, but the analyses of the available data in light of the phylogeny allowed for visualization of the occurrence of karyotypic variations restricted to the clades of the genera *Phyllodytes* and *Osteopilus*. It would be interesting to enlighten the chromosome evolution with other accurate technical approaches and to extend the karyotyping to other species of Lophiohylini, especially new representatives of *Phyllodytes* and *Phytotriades auratus*.

## Appendix

Standard stained meiotic cells Giemsa-stained metaphases I. a. Aparasphenodon bokermanni, 2n = 24; b. Itapotihyla langsdorffii, 2n = 24; c. Trachycephalus sp., 2n = 24; d. T. mesophaeus, 2n = 24; e. T. typhonius, 2n = 24; f. Phyllodytes edelmoi, 2n = 22. g. P. luteolus, 2n = 22. Bar = 10 m. File format: Adobe Acrobat Document (pdf)
