# Supplementary material for: Karyotype analysis of seven species of the tribe Lophiohylini (Hylinae, Hylidae, Anura), with conventional and molecular cytogenetic techniques
Source: Comp Cytogenet. 2012 Dec 3;6(4):409–23. doi: 10.3897/CompCytogen.v6i4.3945 (PMC3834569; doi:10.3897/CompCytogen.v6i4.3945)
Supplement: Standard stained meiotic cells — Giemsa-stained metaphases I. a. Aparasphenodon bokermanni, 2n = 24; b. Itapotihyla langsdorffii, 2n = 24; c. Trachycephalus sp., 2n = 24; d. T. mesophaeus, 2n = 24; e. T. typhonius, 2n = 24; f. Phyllodytes edelmoi, 2n = 22. g. P. luteolus, 2n = 22. Bar = 10 m. File format: Adobe Acrobat Document (pdf) [file CompCytogen-006-409-s001.pdf]

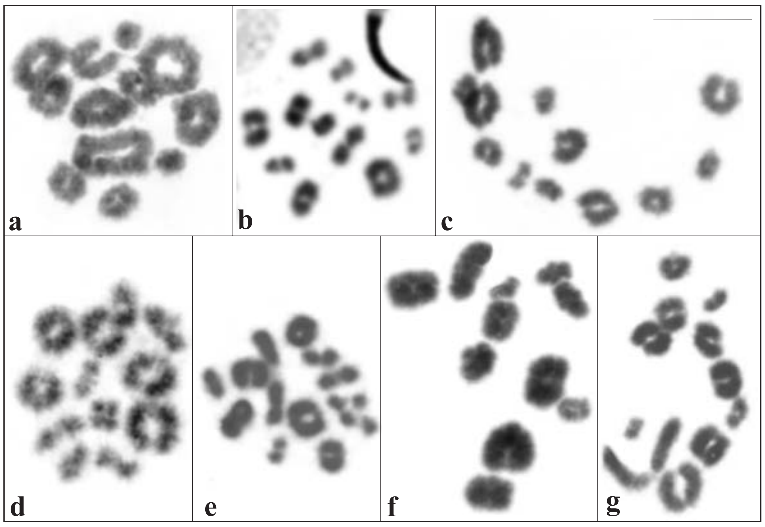

Supplementary file 1. Giemsa-stained metaphases I. **a**. *Aparasphenodon bokermanni*, 2n = 24; **b**. *Itapotihyla langsdorffii*, 2n = 24; **c**. ***Trachycephalus* sp., 2n = 24;** **d**. *T. mesophaeus*, 2n = 24; **e**. ***T. typhonius***, 2n = 24; **f**. *Phyllodytes edelmoi*, 2n = 22. **g**. *P. luteolus*, 2n = 22. Bar = 10 m.
